# The landscape of isoform switches in sepsis: a multicenter cohort study

**DOI:** 10.1038/s41598-022-14231-9

**Published:** 2022-06-17

**Authors:** Lin Chen, Kun Chen, Yucai Hong, Lifeng Xing, Jianjun Zhang, Kai Zhang, Zhongheng Zhang

**Affiliations:** 1 Department of Critical Care Medicine, Affiliated Jinhua Hospital, Zhejiang University School of Medicine, Jinhua, China; 2 Department of Emergency Medicine, Sir Run Run Shaw Hospital, Zhejiang University School of Medicine, Hangzhou, 310016 China; 3 Emergency Department, Zigong Fourth People’s Hospital, 19 Tanmulin Road, Zigong, Sichuan China; 4 Department of Emergency Medicine, Huzhou Central Hospital, Huzhou, 310016 China; 5 Department of Emergency Medicine, Key Laboratory of Precision Medicine in Diagnosis and Monitoring Research of Zhejiang Province, Sir Run Run Shaw Hospital, Zhejiang University School of Medicine, No 3, East Qingchun Road, Hangzhou, 310016 Zhejiang China

**Keywords:** Bacterial infection, Translational research

## Abstract

Sepsis is caused by an uncontrolled inflammatory response, whose underlying mechanisms are not fully understood. It is well known that the majority of human genes can be expressed as alternative isoforms. While isoform switching is implicated in many diseases and is particularly prominent in cancer, it has never been reported in the context of sepsis. Patients presented to the emergency department of three tertiary care hospitals from January 2020 to December 2020 were enrolled. Clinical variables and genome-wide transcriptome of peripheral blood mononuclear cells (PBMC) were obtained. Isoform switching analysis were performed to identify significant isoform switches and relevant biological consequences. A total of 48 subjects with sepsis, involving 42 survivors and 6 non-survivors, admitted to the emergency department of three tertiary care hospitals were enrolled in this study. PBMCs were extracted for RNA sequencing (RNA-seq). Patients (n = 4) with mild stroke or acute coronary syndrome without infection were enrolled in this study as controls. The most frequent functional changes resulting from isoform switching were changes affecting the open reading frame, protein domains and intron retention. Many genes without differences in gene expression showed significant isoform switching. Many genes with significant isoform switches ($|dIF|$> 0.1) were associated with higher mortality risk, including PIGS, CASP3, LITAF, HBB and RUVBL2. The study for the first time described the landscape of isoform switching in sepsis, including differentially expressed isoform fractions between patients with and without sepsis and survivors and nonsurvivors. The biological consequences of isoform switching, including protein domain loss, signal peptide gain, and intron retention, were identified.

## Introduction

Sepsis is a clinical syndrome resulting from an uncontrolled inflammatory response and subsequent organ dysfunction. It is a common disease in the general population that more than 500 cases of sepsis per 100,000 person-years are reported, and the number is increasing^1^. An Intensive Care Over Nations (ICON) audit showed that 30% of patients would have sepsis during their ICU stay, with occurrence rates ranging from 13 to 39%^2,3^. The development of sepsis is associated with a significantly increased risk of mortality. In particular, severe sepsis and septic shock are reported to be associated with mortality rate as high as 50%^4,5^. The cornerstone of the development and progression of sepsis is the imbalance between pro-and anti-inflammatory responses, which has been widely investigated.

Transcriptome profiling has emerged as one of the most powerful approaches for the investigation of sepsis, providing prognostic and predictive utility for the management of sepsis^6^. Transcriptomics contribute to the holistic understanding of sepsis, from clinical to molecular classifications, leading to a more personalized perspective for sepsis diagnostics and interventions^7–9^. However, most of these studies focused on gene expression profiling, ignoring the fact that the expression of alternative isoforms/transcripts of a gene (also known as isoform switching) can result in distinct biological functions. It is well known that the majority of human genes can be expressed as alternative isoforms: approximately 95% of multi-exon genes show evidence of alternative splicing (AS) and approximately 60% of genes have at least one alternative transcription termination site (aTSS)^10,11^. While isoform switching is implicated in many diseases and is especially prominent in cancer^12,13^, it has never been reported in sepsis. Advances in genome-wide RNA sequencing (RNA-seq) combined with the use of versatile tools for the analysis of the resulting short DNA reads have allowed the quantification of transcriptomes at isoform resolution^14^. This advancement has enabled genome-wide analysis of isoform usage and thereby the identification of isoform switching.

The study aimed to describe the landscape of isoform switching in sepsis, including differential isoform fractions evident in patients with and without sepsis, and between survivors and non-survivors. The biological consequences of isoform switching including protein domain loss, signal peptide gain, and intron retention were investigated.

## Methods

### Study setting and participants

The study was conducted in three tertiary care hospitals from January 2020 to December 2020. Patients enrolled in the emergency room were screened for potential eligibility. Patients who fulfilled the following Sepsis-3.0 criteria were enrolled for further evaluation: (1) suspected or documented infection; and (2) acute rise in sequential organ failure assessment (SOFA) score > 2 points^15^. The following exclusion criteria were evaluated: (1) age < 18 years; (2) immunodeficiency including long-term use of corticosteroids, HIV infection, chemotherapy/radiotherapy, or transplantation; (3) advanced malignancy with systemic complications; (4) liver disease with Child–Pugh score > 10 points; (5) pregnancy and (6) patients with a do-not-resuscitate (DNR) order. The study was approved by all participating hospitals and informed consent was obtained from patients or their relatives. Subjects with mild acute coronary syndrome (ACS) and stroke, without signs of infection, were enrolled from the emergency department (ED). ACS patients enrolled had a Global Registry of Acute Coronary Events (GRACE) score < 70 points, and stroke patients had National Institutes of Health Stroke Scale (NIHSS) scores < 4^16,17^. Sepsis was classified according to the primary infection sites: abdomen, the urinary tract, brain, soft tissue, lung, and the intestine and biliary tract.

### Clinical variables and outcomes

Clinical variables including age, sex, comorbidities, weight, height, site of infection, SOFA score, use of mechanical ventilation, C-reactive protein and serum lactate were obtained on day 1 after hospital admission. When multiple measurements were provided for a given variable, the one associated with the worst clinical condition was recorded.

Patients were monitored for all-cause mortality during their hospital stay. Vital status (alive versus dead) at hospital discharge was used as the primary study endpoint. Other outcomes included days of mechanical ventilation (MV) and vasopressor use, defined as the days on using an MV/vasopressor.

### Transcriptome profiling with RNA-Seq

A total of 2 ml of peripheral blood samples were drawn for each patient within 24 h of hospital admission. Peripheral blood mononuclear cells (PBMCs) were isolated, and total RNA was extracted and purified using TRIzol reagent (Invitrogen, Carlsbad, CA, USA) following the manufacturer's procedure. Ribosomal RNA was removed according to the protocol of the Ribo-Zero™ rRNA Removal Kit (Illumina, San Diego, USA). After removing ribosomal RNAs, the remaining RNAs were reverse-transcribed to cDNA,which was then used to synthesize U-labeled double-stranded DNAs with E. coli DNA polymerase I, RNase H and dUTP. Single-or dual-index adapters were ligated to the fragments, and a size selection assay was performed with AMPureXP beads. After heat-labile UDG enzyme treatment of the U-labeled double-stranded DNAs. The ligated products were amplified with PCR under pre-established conditions. The average insert size for the final cDNA library was 300 bp (± 50 bp). Finally, we performed paired-end sequencing on an Illumina NovaSeq™ 6000 following the vendor's recommended protocol.

The length of each read was 150 bp, and each sample generated greater than 10G information. The average number of reads per sample was 88,863,174 (see Additional file 1). First, cutadapt-1.9 (cutadapt.readthedocs.io/en/stable/) was used to remove the reads that contained adapter contamination, low-quality bases and undetermined bases^18^. Then sequence quality was verified using FastQC v0.10.1 (www.bioinformatics.babraham.ac.uk/projects/fastqc/). We used HISAT2-2.0.4 (ccb.jhu.edu/software/hisat2/) to map reads to the genome of *Homo sapiens* obtained from the Ensembl v96 database^19^. The mapped reads of each sample were assembled using StringTie-1.3.4 (ccb.jhu.edu/software/stringtie/) with default parameters^20^. Then, all transcriptomes in the samples were merged to reconstruct a comprehensive transcriptome using the gffcompare tool (github.com/gpertea/gffcompare/). After the final transcriptome was generated, StringTie was used to determine the expression level of the mRNAs by calculating the FPKM (FPKM = [total_exon_fragments/mapped_reads(millions) × exon_length(kb)]). Transcriptome profiling was performed by LC-Biotechnology co.ltd., (Hangzhou, China). The raw data were deposited in the Genome Sequence Archive (https://ngdc.cncb.ac.cn/) under accession: https://ngdc.cncb.ac.cn/gsa-human/browse/HRA002335.

### Isoform switching analysis

Isoform switching in patients with sepsis versus controls and nonsurvivors versus survivors was tested using the DEXSeq method, which searched for bins showing evidence of differential levels of switching between conditions^21,22^. The difference in isoform usage is quantified as the difference in isoform fraction (dIF) calculated as IF_disease-IF_control, and these dIF are used to measure the effect size (like fold changes are in conventional gene/isoform expression analysis). Significant isoform switching was defined as incidence with dIF > 0.1 (i.e. a cutoff > 0.1 is considered as switching by convention) and FDR < 0.05. Genes with log2 fold change > 2 and Q value < 0.05 was considered as differential expression at gene level. The most likely open reading frame (ORF) and the NMD sensitivity of the identified isoforms were then predicted using the genomic coordinates from the transcript model to extract the nucleotide sequence of the transcript from the supplied BS genome object (reference genome). Next, the corresponding protein amino acid sequence of the ORF was obtained simply by translating the nucleotide sequence of the ORF into amino acids.

### Consequences of isoform switching

**Figure 1:**
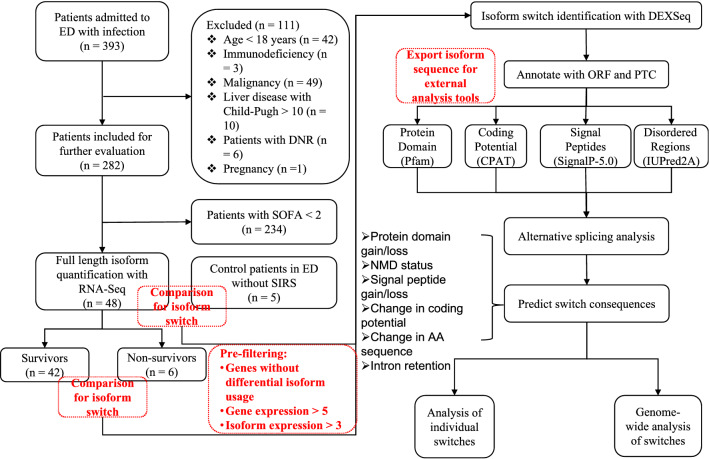
Schematic illustration of the workflow. A total of 393 patients were screened during the study period. After application of the exclusion criteria, 48 patients with sepsis were included for RNA-Seq and subsequent analyses. Isoform switching between patients with and without sepsis and between nonsurvivors and survivors was tested using the DEXSeq algorithm. The sequences of the isoforms were then annotated as ORFs and PTCs. The consequences of isoform switching, such as coding potential, protein domain, intrinsically disordered regions (IDR) and intrinsically disordered binding regions (IDBR), were predicted with external tools. The possibility of alternative splicing was also determined for each case of each isoform switching. Switching can also be analyzed at the individual and genome-wide levels. The gene expression > 5 and isoform expression > 3 was measured in FPKM. *ED* emergency department, *DNR* do not resuscitate, *SOFA* sequential organ failure assessment, *ORF* open reading frame, *PTC* pre-mature termination codons, *NMD* Nonsense Mediated Decay.

Since we knew the sequence of the identified isoforms, we proceeded to predict the coding potential^23^, protein domains^24^, signal peptide^25^, intrinsically disordered regions (IDR) and intrinsically disordered binding regions (IDBR) using an external sequence analysis tools including Pfam, CPAT, SignalP-5.0, and IUPred2A^26^. Alternative splicing including exon skipping, intron retention, alternative 3′ splice sites, alternative 5′ splice sites, and mutually exclusive exons was annotated^27^. A schematic illustration of the workflow is shown in Fig. 1. The isoform switching analyses were performed using the IsoformSwitchAnalyzeR (version 1.14.1)^28^.

### Quantitative real-time PCR validation

FLOT2 was used for qPCR validation because it showed a large dIF between sepsis and controls and the difference was consistently observed across different sepsis types. Quantitative Real-time PCR (qRT-PCR) was performed using qTOWER2.2 (Germany). The primer pairs are listed as follows: ENST00000394908: F:ACCAGAGGACCCAGAGAA; R:CCTTCTCCGTCTGTCTTCC, ENST00000585169: F:GGACCCGACACCAGAGAC; R:GGTTCCTTCTCCGTCTGTCT, MSTRG.31878.2: F:AGGGCAGAGAAGGACAAA; R:TGTCTTTCCGCTGTTTGG. Following an initial 3 min denaturation/activation step at 95 °C, the mixture was subjected to 40 cycles of amplification (denaturation for 10 s at 95 °C, annealing and extension for 30 s at 58 °C). The specificity of the qRT-PCR reaction for each amplified product was verified by melting curve analysis (60–95 °C, + 1 °C /cycle, holding time 4 s). GAPDH was used as an internal control gene. Similar validations were performed for genes ALDH3B1 (ENST00000342456 and ENST00000615368) and MEGF9 (MSTRG.75081.6 and MSTRG.75081.8).

### Alternative splicing as identified by rMATS

Since there are multiple tools available for the analysis of alternative splicing employing RNA-Seq data, we validated previous findings with the rMATS tool (version: 4.0.2)^29^. rMATS employs a hierarchical model to simultaneously address variability among replicates and sampling uncertainty in individual replicates. The alternative splicing events were compared between sepsis and control groups. The overlap genes associated the same AS events were compared between the two methods.

### Statistical analysis

Baseline characteristics were compared between survivors and non-survivors. Continuous variables were expressed as mean and standard deviation for data of normal distribution, and median and interquartile range for skewed data. Continuous variables were compared using student t test or rank sum test as appropriate. Categorical variables were expressed as the number and percentage, and Chi-square test was used for between group comparisons. The intersection of genes common to the sepsis types was explored using upset plot by UpSetR package (version: 1.4.0). Statistical significance was considered when two-tailed p was less than 0.05. Adjusted p value (here we refer to q value) was reported to consider the false discovery rate (FDR). All analyses were performed in R (version: 4.1.1).

### Ethics approval and consent to participate

The study was approved by the ethics committee of Sir Run Run Shaw hospital (20201014–39) and written informed consents were obtained from patients or their relative kins. All methods were carried out in accordance with relevant guidelines and regulations.

## Results

### Clinical characteristics of the patients

A total of 393 patients were screened during the study period. After application of the exclusion criteria, 48 patients with sepsis were included for RNA-Seq and subsequent analysis. Five patients without inflammatory response syndrome were included as controls (Fig. 1). The mean age of the included patients was 71.7 ± 13.5 years (Table 1). The most common infection site was the lung (29%), followed by the abdomen (23%) and urinary tract (19%). Survivors were less likely to have hypertension than nonsurvivors (6/6 vs. 36/42; p = 0.004). Nonsurvivors showed significantly more MV days than survivors (17.5 (6.3, 26.5) vs. 1.5 (0, 7.0) days; p = 0.042, Table 1).

**Table 1:** Clinical characteristics of the sepsis cohort.

Variables	Total (n = 48)	Survivors (n = 42)	Non-survivors (n = 6)	p*
Age (years), mean ± SD	71.69 ± 13.50	71.21 ± 14.12	75.00 ± 7.95	0.355
Sex, male (%)	29 (60)	24 (57)	5 (83)	0.381
**Infection site, n (%)**				0.741
Abdomen	11 (23)	9 (21)	2 (33)	
Biliary	3 ( 6)	3 ( 7)	0 ( 0)	
Brain	2 ( 4)	2 ( 5)	0 ( 0)	
Intestine	4 ( 8)	4 (10)	0 ( 0)	
Lung	14 (29)	11 (26)	3 (50)	
Soft tissue	5 (10)	4 (10)	1 (17)	
Urinary	9 (19)	9 (21)	0 ( 0)	
SOFA, mean ± SD	6.38 ± 2.82	6.19 ± 2.87	7.67 ± 2.16	0.174
Height (cm), mean ± SD	165.70 ± 8.27	165.50 ± 8.58	167.20 ± 5.89	0.587
Weight (kg), median (Q1, Q3)	60 (55, 65)	60 (55, 65)	60 (60, 65)	0.687
Diabetes, n (%)	10 (21)	9 (21)	1 (17)	1.000
Hypertension, n (%)	21 (44)	15 (36)	6 (100)	0.004
Cardiac failure, n (%)	8 (17)	6 (14)	2 (33)	0.258
MV, n (%)	22 (46)	19 (45)	3 (50)	1.000
Lactate (mmol/L), median (Q1, Q3)	2.30 (1.50, 4.25)	2.30 (1.50, 4.03)	2.85 (1.68, 4.40)	0.779
MV (days), median (Q1, Q3)	3.00 (0.00, 9.25)	1.50 (0.00, 7.00)	17.50 (6.25, 26.50)	0.042
Vasopressor (days), median (Q1, Q3)	2.00 (0.00, 5.00)	2.00 (0.00, 4.75)	7.00 (2.75, 12.00)	0.051

*SD* standard deviation, *SOFA* sequential organ failure assessment, *MV* mechanical ventilation, *Q1* the first quartile, *Q3* the third quartile, *CRP* C-reactive protein.

*p value was reported for the comparison between survivors and non-survivors.

### Isoform switching in patients with sepsis versus controls

**Figure 2:**
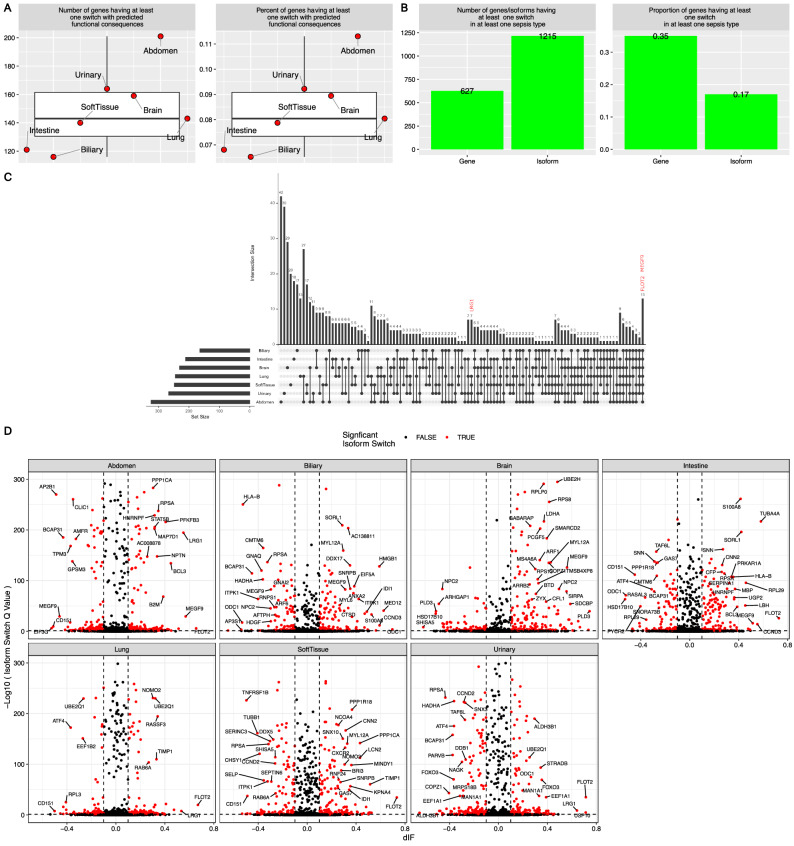
Isoform switching with predicted consequences in patients with sepsis versus controls. (**A**) Extent of isoform switching in different sites of infection. Left, the number of genes undergoing at least one isoform switching in the patients with sepsis compared to that in the controls and predicted to have a functional consequence in each sepsis type. The right panel shows the corresponding fractions of analyzed genes that underwent at least one isoform switching episode. (**B**) The Extent of isoform switching associated with each sepsis type. Bar plots showing the number and proportion of tested genes and isoforms involved in isoform switching with predicted functional consequences associated with at least one sepsis type. (**C**) UpSet plot showing the intersection of genes common to the sepsis types. The bar plot indicates the intersection size (number of genes). For example, the first bar shows that 51 genes underwent at least one significant isoform switch that was unique to abdominal sepsis, and 16 genes underwent at least one significant isoform switch that was common to the lung and abdomen. (**D**) Significant isoform switching as defined by FDR and dIF across all types of sepsis compared to that in controls. Genes with |dIF|> 0.25 and q value < 0.001 were labeled by gene names, and those with |dIF|> 0.1 and q value < 0.05 were labeled by red color. *dIF* difference in the isoform fraction; *FDR* false discovery rate.

**Figure 3:**
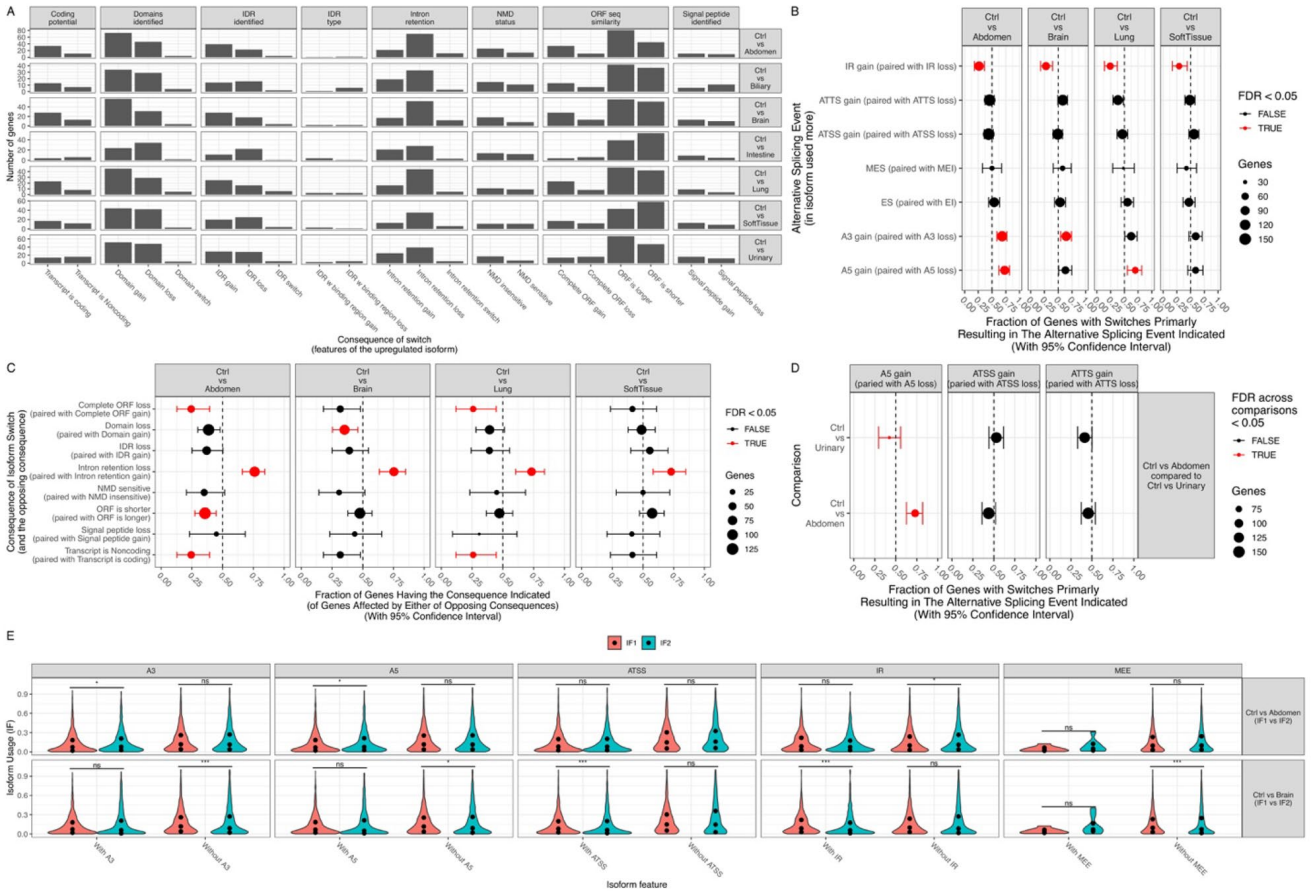
Consequences of isoform switching across different types of sepsis. (**A**) The number of isoform switching consequences associated with different sepsis types. (**B**) Enrichment analysis exploring the uneven distribution of opposite alternative splicing events. The fraction of genes with switching primarily resulting in an alternative splicing event is indicated. (**C**) Enrichment analysis exploring the uneven distribution of opposite switching consequences. (**D**) Comparison of differences in the fraction of alternative splicing events in patients with abdominal sepsis compared to those with urinary tract sepsis. (**E**) Isoform expression between the patients with sepsis of the abdomen and brain and the controls. *dIF* difference in the isoform fraction; *FDR* false discovery rate; *IR* intron retention, *IDR* intrinsically disordered regions, *NMD* nonsense-mediated decay, *ORF* open reading frame, *IR* intron retention. *A5* alternative 5′ donor site (changes in the 5′ end of the upstream exon) , *A3* alternative 3′ acceptor site (changes in the 3′ end of the downstream exon) , *ATSS* alternative transcription start site, *ATTS* alternative transcription termination site, *ES* exon skipping, *MES* multiple exon skipping, skipping > 1 consecutive exon, *MEE* mutually exclusive exons.

The case of isoforms switching compared to non-sepsis controls varied across sepsis types (classified by infection site). Patients with abdominal infections exhibited the largest number of genes undergoing isoform switching with functional consequences, as well as the fraction of genes passed filtering criteria for isoform switching, followed by patients with urinary tract, brain, and lung infections (Fig. 2A,B). The number of genes with significant isoform switching with predicted functional consequences that were common to all sepsis types was very small, indicating heterogeneity across sepsis types (Fig. 2C). Compared to the controls, the patients with sepsis consistently showed significant isoform switching of the FLOT2, LRG1 and MEGF9 genes (Fig. 2D). The number of genes with isoform switching consequences was generally consistent across sepsis types. However, there was also heterogeneity across sepsis types: patients with intestinal sepsis had fewer isoform switching genes with coding potential, and patients with lung sepsis showed greater IR loss (Fig. 3A). There was less IR gain than loss in patients with abdomen, brain, lung and soft tissue sepsis. There were more A3 and A5 gains in the patients with abdominal sepsis than in the controls (Fig. 3B). The four types of sepsis were consistently associated with more IR loss than gain. Furthermore, there was a significant difference in A5 gain between the patients with urinary tract and those with abdominal sepsis, with patients with abdominal sepsis more likely to show A5 gain, and those with urinary tract sepsis were less likely to have A5 gain. There was significant uneven distribution of opposite switching consequences (Fig. 3C). Abdomen sepsis was more likely to have A5 gain than the urinary sepsis (Fig. 3D,E).

**Figure 4:**
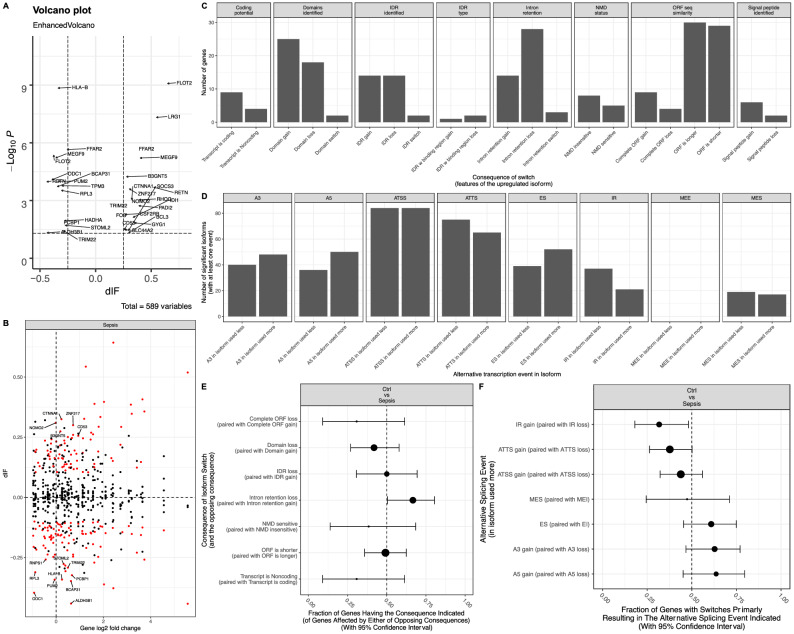
Isoform switching between patients with sepsis and controls as determined by pooling all data of sepsis types together. (**A**) Volcano plot shows differentially expressed isoforms, and the genes with significant p values and dIFs are labeled with gene names. The dashed lines indicate significance values for a dIF = 0.1 and FDR = 0.05. (**B**) Comparison between gene expression and dIF. Red indicates genes with significant isoform switching. Genes without significant fold changes but with significant dIF are labeled with gene names. (**C**) Consequences of isoform switching between the patients with sepsis and the controls. (**D**) Alternative splicing events in isoforms. (**E**) Genome-wide enrichment of specific consequences for each set of opposing consequences (e.g., domain gain vs loss) by analyzing the fraction of events associated with each consequence. IR loss is significantly more likely to occur than IR gain. (**F**) Enrichment of specific splice events for each set of opposing events (e.g., IR gain vs loss) as determined by analyzing the fraction of events associated each type of consequence. *dIF* difference in the isoform fraction, *FDR* false discovery rate, *IR* intron retention, *IDR* intrinsically disordered regions, *NMD* nonsense-mediated decay, *ORF* open reading frame, *IR* intron retention. *A5* alternative 5′ donor site (changes in the 5′ end of the upstream exon), *A3* alternative 3′ acceptor site (changes in the 3′ end of the downstream exon), *ATSS* alternative transcription start site, *ATTS* alternative transcription termination site, *ES* exon skipping, *MES* multiple exon skipping, skipping > 1 consecutive exon, *MEE* mutually exclusive exons.

Isoform switching was further explored by combining all sepsis types together (Additional File 2). Genes with different isoforms included FLOT2, LRG1, HLA-B and MEGF9 (Fig. 4A). The relationship between isoform switch and differential expression was explored. Many genes without differences in gene expression showed significant isoform switching (Fig. 4B). The most frequent changes were those affecting ORFs, protein domains and IR (Fig. 4C,D). Notably, the opposite consequences were not evenly distributed, e.g., more IR loss than IR gain (Fig. 4E). There were fewer IR and ATTS gains in the patients with sepsis than in the controls (Fig. 4F). After FDR correction of these comparisons, none of the differences were significant.

### Validation with qPCR experiments

**Figure 5:**
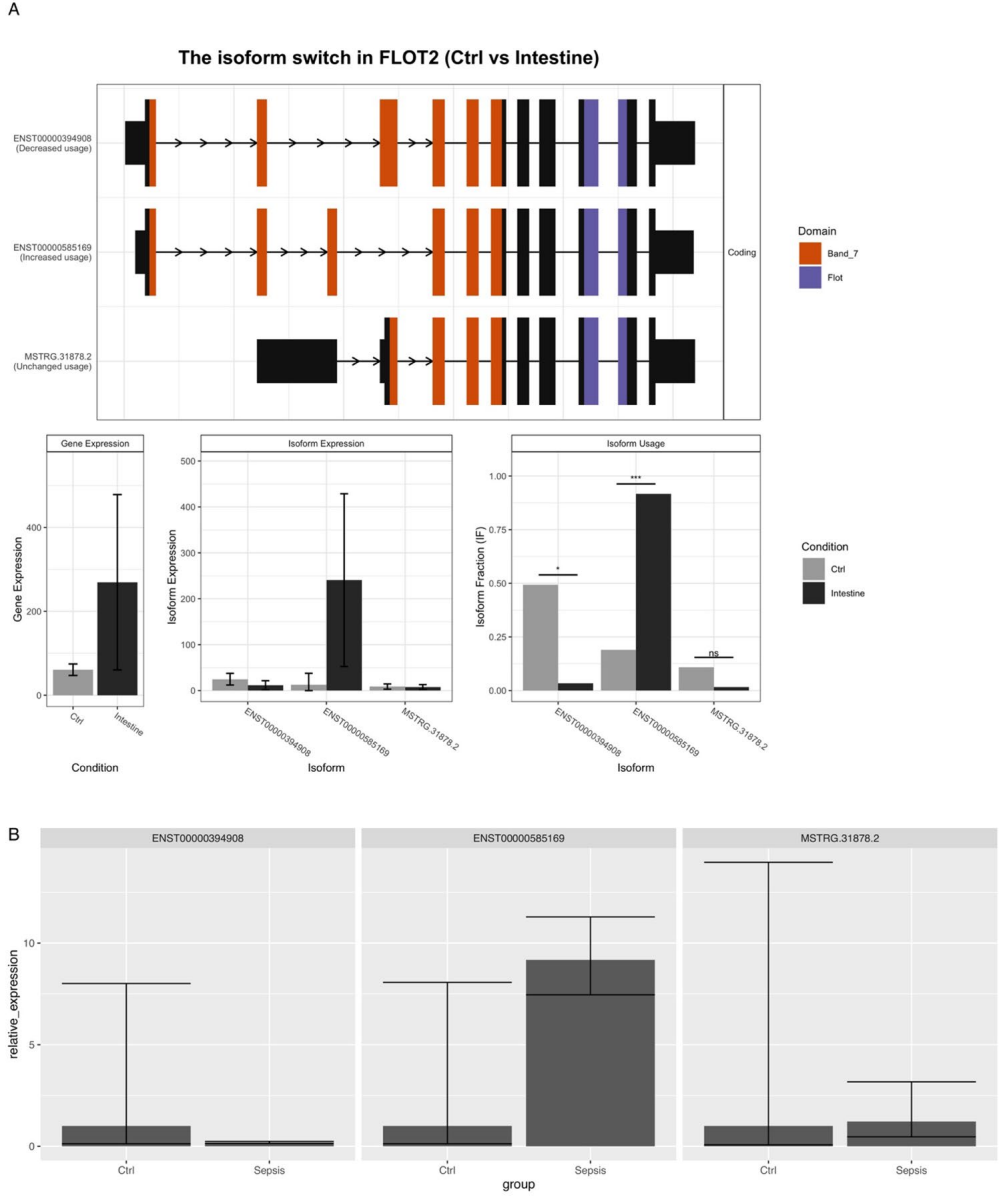
Isoform switching in the FLOT2 gene and qPCR validation. (**A**) Isoform switching in the FLOT2 gene was identified by RNA-Seq. Isoform ENST00000394908 and ENST00000585169 expression was significantly different between the patients with intestinal sepsis and the controls. (**B**) The qPCR results confirmed that transcript ENST00000585169 was upregulated in the sepsis samples, with significant differential isoform fraction expression (p < 0.05).

Transcripts with considerable abundance were validated with qPCR experiments. Three transcripts (MSTRG.31878.2, ENST00000394908, ENST00000585169) of the FLOT2 gene were quantitatively measured with qPCR to obtain Ct values for each sample. The $\Delta \Delta {C}_{t}$ method was used to estimate the normalized relative expression of target genes. The results showed that the transcript ENST00000585169 was significantly increased in the patients with sepsis compared to the controls, with significant differential isoform expression (p < 0.05; Fig. 5). The qPCR validation was also performed for genes ALDH3B1 (relative expression for ENST00000342456 [10.2 $\pm$ 3.2 vs. 5.5 $\pm$ 3.1; p < 0.05 for control vs. sepsis] and ENST00000615368 [2.2 $\pm$ 0.2 vs. 10.5 $\pm$ 5.1; p < 0.05 for control vs. sepsis]) and MEGF9 (MSTRG.75081.6 [5.2 $\pm$ 3.1 vs. 2.5 $\pm$ 1.1; p < 0.05 for control vs. sepsis] and MSTRG.75081.8 [1.2 $\pm$ 0.2 vs. 5.8 $\pm$ 2.1; p < 0.05 for control vs. sepsis]), which confirmed the results of RNA-seq data analysis (Additional file 3).

### Isoform switching in nonsurvivors versus survivors

**Figure 6:**
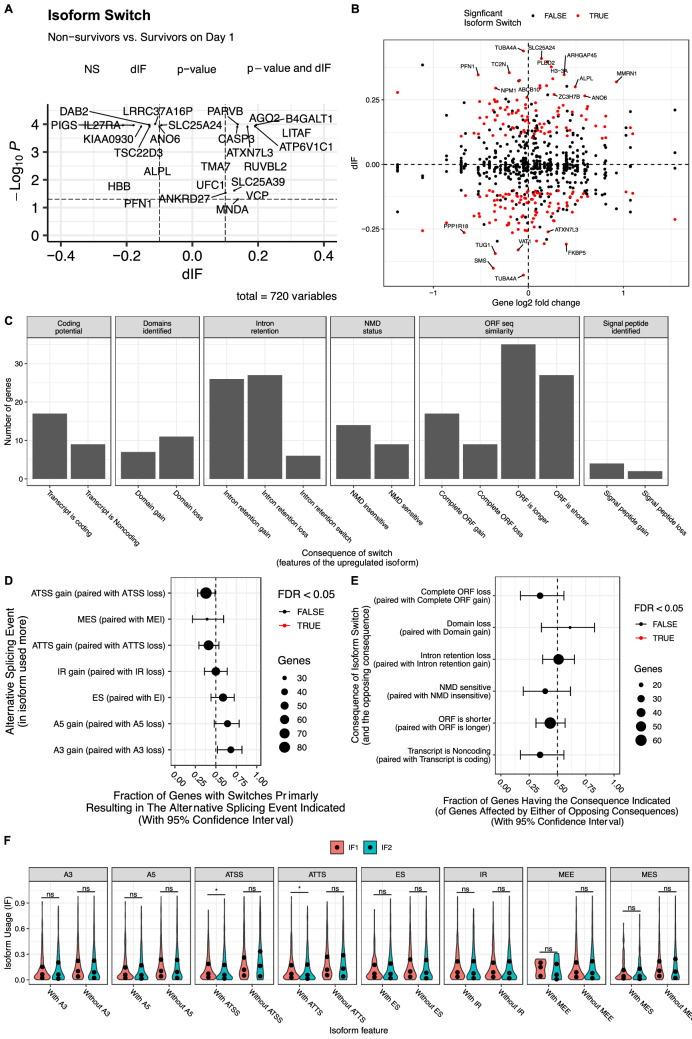
Isoform switching between nonsurviving and surviving patients with sepsis. (**A**) Volcano plot shows differentially expressed isoforms, and the genes with significant p values and dIFs are labeled with gene names. The dashed lines indicate significance values for dIF = 0.1 and FDR = 0.05. (**B**) Comparison between gene expression levels and dIF. Genes without significant fold changes but with significant dIF are labeled with gene names. (**C**) Consequences of isoform switching between nonsurvivors and survivors. (**D**) Alternative splicing events in isoforms relying on the identification of pairs of isoforms involved in the switch. (**E**) Genome-wide enrichment of specific consequences for each set of opposing consequences (e.g., domain gain vs loss) by analyzing the fraction of events associated with one consequence. There was no statistically significant difference between survivors and nonsurvivors. (**F**) Analysis of alternative splicing from the perspective of the individual splice type. *IF1* isoform fraction for survivors, and *IF2* isoform fraction for nonsurvivors.

Isoform switching between nonsurvivors and survivors was explored (Additional file 4). Many genes with significant isoform switching ($|dIF|$> 0.1), including PIGS, CASP3, LITAF, HBB and RUVBL2, were associated with the increased risk of mortality (Fig. 6A). Notably, many genes without differential gene expression showed significant isoform changes, such as TUBA4A, SLC25A24, ARHGAP45 and TC2N (Fig. 6B). The most frequent changes affected ORF, IR and IDR (Fig. 6C). Nonsurvivors displayed a higher fraction of genes with ATSS loss than ATSS gain (54/33; 0.38; 0.28 to 0.49; p = 0.03; Table 2); there were more A3 gains than losses in nonsurvivors (28/13; 0.68; 0.52 to 0.82; p = 0.03; Fig. 6D). However, these differences did not reach statistical significance after FDR adjustment. The fraction of genes undergoing specific isoform switching, as determined by the consequences of switching, was similar between survivors and nonsurvivors (Fig. 6E). The isoform expression of ATSS (0.073 vs. 0.058; q = 0.021) and ATTS (0.070 vs. 0.057; q = 0.045; Table 3) was significantly higher in survivors than in nonsurvivors (Fig. 6F).

**Table 2:** The number of specific splice events and associated opposing events.

AS type	nUp	nDown	propUp	p value	Q value
A3 gain (paired with A3 loss)	28	13	0.68 (0.52–0.82)	0.03	0.13
A5 gain (paired with A5 loss)	27	15	0.64 (0.48–0.78)	0.09	0.24
ATSS gain (paired with ATSS loss)	33	54	0.38 (0.28–0.49)	0.03	0.13
ATTS gain (paired with ATTS loss)	28	40	0.41 (0.29–0.54)	0.18	0.36
ES (paired with EI)	30	21	0.59 (0.44–0.72)	0.26	0.42
IR gain (paired with IR loss)	27	27	0.50 (0.36–0.64)	1.00	1.00
MEE gain (paired with MEE loss)	0	1	0.00 (0.00–0.98)	1.00	1.00
MES (paired with MEI)	11	17	0.39 (0.22–0.59)	0.34	0.46

The number of genes with a specific AS type is obtained by comparing non-survivors versus survivors. For examlpe, there were 28 genes filtered by significance test with A3 gain and 13 genes with significant A3 loss.

*nUp* number of up regulation, *nDown* number of downregulation, *propUp* proportion of upregulation, *AS* alternative splicing, *IR* intron retention, *A5* alternative 5′ donor site (changes in the 5′end of the upstream exon) , *A3* Alternative 3′ acceptor site (changes in the 3′end of the downstream exon), *ATSS* alternative transcription start site, *ATTS* alternative transcription termination site, *ES* exon skipping, *MES* multiple exon skipping, skipping of > 1 consecutive exons, *MEE* mutually exclusive exons.

**Table 3:** Genome wide Analysis of alternative splicing.

Category	Isoform feature	n	Median IF in survivors	Median IF in nonsurvivors	Median DIF	Wilcox p	Wilcox Q
A3	With A3	266	0.063	0.063	0.000	0.276	0.441
A3	Without A3	454	0.102	0.088	− 0.014	0.025	0.066
A5	With A5	259	0.059	0.056	− 0.004	0.349	0.462
A5	Without A5	461	0.104	0.092	− 0.012	0.023	0.066
ATSS	With ATSS	529	0.073	0.058	− 0.015	0.001	0.021
ATSS	Without ATSS	191	0.118	0.163	0.045	0.487	0.519
ATTS	With ATTS	477	0.070	0.057	− 0.013	0.006	0.045
ATTS	Without ATTS	243	0.119	0.132	0.013	0.709	0.709
ES	With ES	287	0.072	0.069	-0.003	0.363	0.462
ES	Without ES	433	0.098	0.083	− 0.016	0.022	0.066
IR	With IR	209	0.088	0.076	− 0.012	0.172	0.306
IR	Without IR	511	0.088	0.080	− 0.009	0.051	0.102
MEE	With MEE	8	0.149	0.060	− 0.089	0.382	0.462
MEE	Without MEE	712	0.088	0.079	− 0.009	0.022	0.066
MES	With MES	179	0.043	0.044	0.001	0.404	0.462
MES	Without MES	541	0.107	0.096	− 0.011	0.037	0.084

The significance test is performed using Mann–Whitney-U and resulting p-values are corrected using FDR (Benjamini-Hochberg).

*IF* isoform fraction, *AS* alternative splicing, *IR* intron retention. *A5* Alternative 5′ donor site (changes in the 5′end of the upstream exon), *A3* alternative 3′ acceptor site (changes in the 3′end of the downstream exon), *ATSS* alternative transcription start site, *ATTS* alternative transcription termination site, *ES* exon skipping, *MES* multiple exon skipping, skipping of > 1 consecutive exons, *MEE* mutually exclusive exons.

### Alternative splicing as identified by rMATS

**Figure 7:**
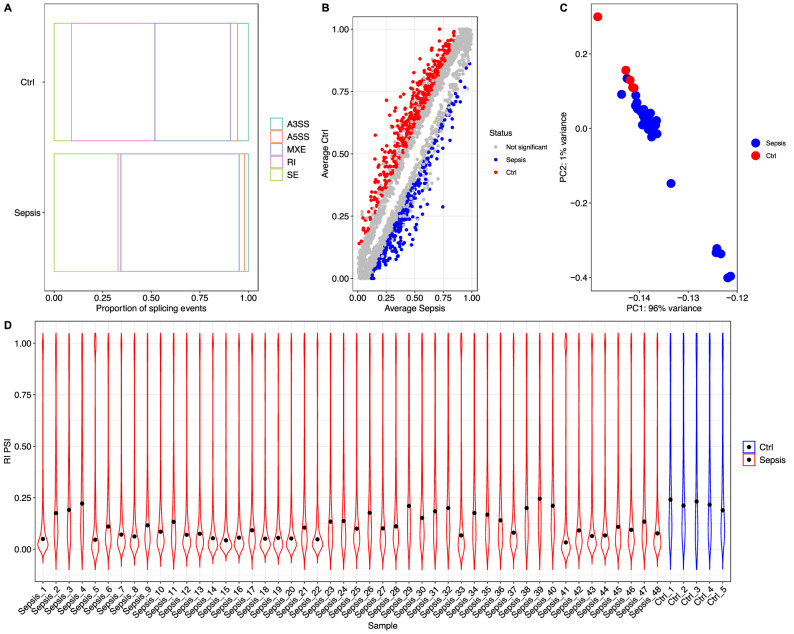
Alternative splicing analyzed by rMATS. (**A**) Distribution of the proportion of alternative splicing events. MXE account for a larger proportion of AS events in the patients with sepsis than in the controls. (**B**) Dot plot showing significantly different events involving MXE. The vertical and horizontal axes are the average PSI for the control and sepsis groups, and each dot represents one gene. (**C**) PCA plot showing the first two principal components of the PSI for an event involving MXE. The sepsis and control groups were easily separated by MXE. (**D**) The distribution of RI with the PSI for each sample. The control group showed higher values than the sepsis group. *MXE* mutually exclusive exons, *RI* retained intron, *AS* alternative splicing, *PSI* percent spliced in, *PCA* principal component analysis.

Since there are multiple tools available for the analysis of alternative splicing employing RNA-Seq data, we validated previous findings with the rMATS tool. The two methods, DEXSeq and rMATS, used in our study represent the exon- and event-based methods, respectively^30^. The results showed that alternative splicing events were significantly different between the sepsis and control groups. The mutually exclusive exon (MXE) event affected a larger proportion of patients in the sepsis group than in the control group (Fig. 7A). Numerous genes showed statistically significant differences in MXE between the sepsis and control groups (Fig. 7B). The PSI score was able to distinguish between sepsis and controls (Fig. 7C,D). Many overlapping genes were associated the same AS events, as determined by the DEXseq and the rMATS methods (Table 4).

**Table 4:** Common genes with significant alternative splicing events identified by both rMATS and DEXseq.

Type of AS	Gene names
A3	TBC1D10C, MAP2K3, MXD1, EDF1
A5	RPLP0, BCL3
ES	PFKFB3, FAM107B, MS4A4A, RTN3, ARHGDIB, LRRK2, LAMP1, BCAP31, FLOT2, ACTN4, PUM2, FGR, PHC2, ZNF217, UQCRC1, MARCHF6, CD53, ATP1A1, TPM3, FAM214B, RBM3
IR	IDI1, TRIM22, PPP1CA, LRRK2, CALCOCO1, LAMP1, SLC44A2, HADHA, NCF4, DDX17, CTNNB1, UQCRC1, HLA-C, MKRN1, ZYX, GPAA1, RBM3
MES	PFKFB3, NCOA4, ANO6, MAP2K3, TNFRSF1B, FGR, PHC2, RASSF2, GLB1, CTNNB1, GYG1, CD53, MKRN1, TPM3, TLN1, STOM, FCGR3B, ARF1

*AS* alternative splicing, *ES* exon splicing, *IR* intron retention, *MES* mutually exclusive splicing.

### Differential gene expression between the sepsis and control groups

**Figure 8:**
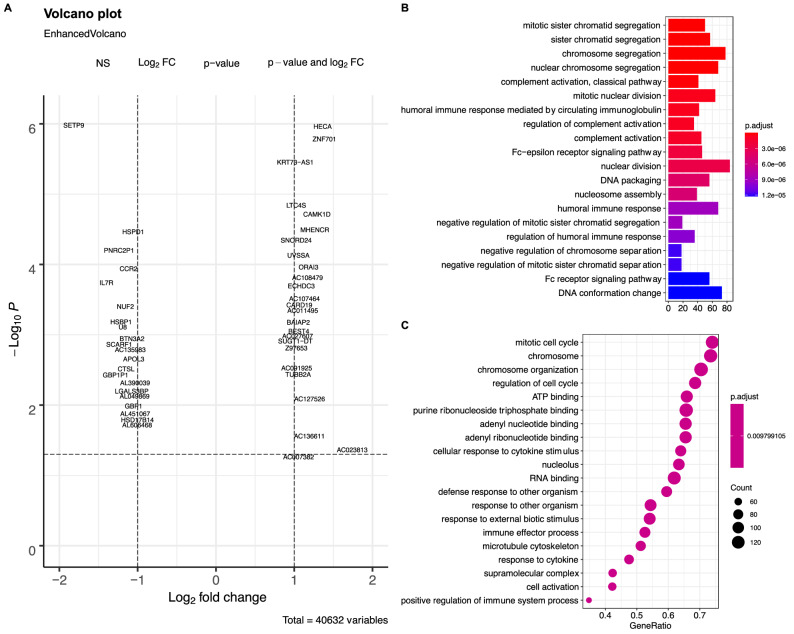
Differential gene expression between the sepsis and control groups. (**A**) Volcano plot showing differentially expressed genes between the sepsis and control groups. (**B**) GO enrichment analysis with the overrepresentation method. (**C**) Gene set enrichment analysis with GO terms.

Differential gene expression analysis was performed using the DESeq2 method^31^. Many genes were differentially expressed between the sepsis and control groups, such as HECA, ZNF701, KRT7 $\beta$-AS1 and SETP-9 (Fig. 8A). These genes were significantly enriched in pathways such as complement activation, humoral immune response mediated by circulating immunoglobulin and cellular response to cytokine stimulus (Fig. 8B,C).

## Discussion

This study is the first to report genome-wide isoform switching in patients with sepsis. Our comparative analyses of patients with sepsis versus controls, as well as survivors versus nonsurvivors, revealed many isoform switching events. Many of these isoform switches were predicted to have functional consequences. Interestingly, many genes in the same dataset exhibiting significant isoform switching were not detected by conventional DGE analysis. This outcome can be explained either by the isoforms with low expression levels or antagonistic, inverse changes in other isoforms of the same gene, canceling out the net change at the gene expression level. Another interesting finding was that isoform switching showed substantial heterogeneity across sepsis subtypes as defined by infection sites. Some alternative splicing events were significantly different between patients with different subtypes of sepsis, whereas these differences were nullified when all the sepsis samples were pooled together.

Our findings suggest that isoform switching events in sepsis may have important downstream consequences, such as domain gain, IDR gain, NMD insensitivity and ORF gain. Irrespective of the total gene expression levels, the changes in the relative expression of different isoforms of a gene affect the ratio of the resulting protein isoforms and thus may influence biological processes^32,33^. Moreover, our study shows that a substantial number of switches occur between protein coding and noncoding transcript isoforms, thereby affecting the overall protein level.

To the best of our knowledge, no study has explored genome-wide isoform switching in sepsis. However, the landscape of isoform switching has been explored in oncology and Parkinson's disease (PD) studies^34^. In sepsis, our study showed a substantially greater number of significant isoform switching events than that had been reported for PD. In a previous study, only 19 genes (23 switching events) showed significant isoform switching in PD samples^32^. In our study, the number of genes involved in sepsis was much greater, and 1215 isoforms of 627 genes were found to undergo least one switch in association with least one sepsis type. Most likely, isoform switching is a rapid acute phase response to pathogens, and this response plays an important role in controlling pathogen invasion and inflammatory responses. PD and tumors are chronic disorders in which differential isoform expression plays a less important role in pathogenesis. The proportion of genes with isoform switching has been reported to be similar in sepsis (17%) and cancer (19%)^13^, indicating that isoform switching with potential functional consequences were common.

In our study, we identified several genes with significant isoform switching that have never been associated with sepsis. FLOT2 encodes the protein Flotillin-2 and may act as a scaffolding protein within caveolar membranes, functionally participating in the formation of caveolae or caveolae-like vesicles. It plays important roles in epidermal cell adhesion and epidermal structure and function in several cancers^35–37^. More recently, flotillin was found to play an essential role in protective antifungal immunity^38^. In our study, we found that FLOT2 abundance was significantly increased in the patients with sepsis compared to the controls. Three isoforms expressed led to functional consequences. The isoform fraction of ENST00000585169 was increased, while the isoform expression of ENST00000394908 was decreased, suggesting important roles of ENST00000394908 in the pathogenesis of sepsis. Isoform switching might result in a domain change in the FLOT2 protein, as predicted by the bioinformatics approach used in our study. MEGF9, a transmembrane protein with multiple EGF-like repeats, is associated with the nervous system^39^. Our study found its potential role in the pathogenesis of sepsis, making it a target for further exploration. LRG1 is associated with the innate immune system and can be further explored for its role in the pathogenesis of sepsis^40^.

Our study showed that isoform switching in patients with sepsis compared to controls and survivors compared to nonsurvivors led significant biological consequences, affecting the open reading frame, protein domain, or intron retention. A protein domain is a region of in the polypeptide chain that is self-stabilizing and that folds independently from the remainder of the protein. Each domain forms a compact folded three-dimensional structure. Many proteins consist of several domains. One domain may appear in a variety of different proteins. The loss or gain of one domain during the development of sepsis may alter the biological function of the encoded protein. Our study found that there were approximately 25 gene isoforms that led to domain gain and 18 genes with isoforms that led to domain loss. However, the number of domain gains versus domain losses was not significantly different.

Several limitations must be acknowledged in the current study. First, the study enrolled patients with mild stroke or ACS to serve as control subjects. They were chosen because many patients with these conditions are in emergency rooms, and it can be difficult to obtain biospecimens from healthy adults. The size of the control group was very low and that a larger number of controls may change the results. Second, the patients presented with a variety of causes, and the heterogeneity significantly reduced the statistical power for identifying less abundant transcripts. However, we stratified sepsis into subtypes according to the source of infection. The results confirmed that many isoforms were differentially expressed in the patients with sepsis compared to the controls. Third, although we tried to obtain blood samples in the emergency department, the time from sepsis onset to ED arrival was difficult to determine. Some evidence suggests that the transcriptome profile can be different at different time points^41^. However, we made every effort to minimize the impact of the timing of sepsis by enrolling patients immediately after ED arrival, not waiting until ICU admission. The length of stay in the ED is usually long in China^42^, and this prolonged stay may have further confounded the transcriptome analysis. Forth, consecutive measurement of isoform abundance at baseline, acute phase and recovery phase during sepsis course can help to control between-subjects confounding factors. However, it is difficult to obtain samples from the same patient in a time-series manner. Thus, the study was designed to answer the question on whether there was difference between sepsis versus non-sepsis. Another limitation is that the patients were recruited only from people with Chinese ethnicity. The outcomes may be different in other ethnic populations.

## Conclusion

The study for the first time described the landscape of isoform switching in sepsis, including differentially expressed isoform fractions between patients with and without sepsis and survivors and nonsurvivors. The biological consequences of isoform switching, including protein domain loss, signal peptide gain, and intron retention, were identified.

## Supplementary Information

Supplementary Information 1.

Supplementary Information 2.

Supplementary Information 3.

Supplementary Information 4.

## Data Availability

The raw data were deposited in the Genome Sequence Archive (https://ngdc.cncb.ac.cn/) under accession https://ngdc.cncb.ac.cn/gsa-human/browse/HRA002335.

## Abbreviations

MXE: Mutually exclusive exons
RI: Retained intron
AS: Alternative splicing
PSI: Percent spliced in
PCA: Principal component analysis
dIF: Difference in the isoform fraction
FDR: False discovery rate
IR: Intron retention
IDR: Intrinsically disordered regions
NMD: Nonsense-mediated decay
ORF: Open reading frame
A5: Alternative 5′ donor site (changes in the 5′ end of the upstream exon)
A3: Alternative 3′ acceptor site (changes in the 3′ end of the downstream exon)
ATSS: Alternative transcription start site
ATTS: Alternative transcription termination site
ES: Exon skipping
MEE: Mutually exclusive exons
