# Supplementary material for: The landscape of isoform switches in sepsis: a multicenter cohort study
Source: Sci Rep. 2022 Jun 17;12:10276. doi: 10.1038/s41598-022-14231-9 (PMC9205547; doi:10.1038/s41598-022-14231-9)
Supplement: Supplementary file 3 — Supplementary Information 3. [file 41598_2022_14231_MOESM3_ESM.docx]

Additional file 4

**Figure e1. Isoform switching in the ALDH3B1 gene**

Isoform switching in the ALDH3B1 gene was identified by RNA-Seq. Isoform ENST00000342456 and ENST00000615368 expressions were different between lung sepsis and the controls.

**Figure e2. Isoform switching in the MEGF9 gene**

Isoform switching in the MEGF9 gene was identified by RNA-Seq. Isoform MSTRG.75081.6 and MSTRG.75081.8 expressions were different between lung sepsis and the controls.
